# AN ACADEMIC-COMMUNITY PARTNERSHIP FOR IMPROVING ORAL HEALTH OF ADULTS 75-PLUS YEARS IN RURAL SOUTHEASTERN OREGON

**DOI:** 10.1093/geroni/igz038.527

**Published:** 2019-11-08

**Authors:** tamara rose, darcy mize

**Affiliations:** Oregon Health & Science University Regional Campus, Klamath Falls, Oregon, United States

## Abstract

This presentation describes a series of research projects undertaken by a school of nursing to develop and test a model for interprofessional (IPE) practice and education. A pilot study and two funded projects have been completed and one funded project is currently underway. A goal has been to build an academic-community partnership to address health disparities for people 75 years and older living in rural areas of the state. This population is growing and more apt to be living in poverty. Low oral health literacy and limited dental health services in rural areas contribute to the likelihood of oral health problems impacting overall health. IPE supports the development of teamwork to improve health outcomes for patients by raising awareness about the relationship of oral health to overall health. This evolving IPE model has joined baccalaureate nursing and dental hygiene students in shared learning and practice. After classroom and simulation-based learning, student teams provided screening and education in primary care settings. The current study has expanded team membership by adding medical, dental and/or nutrition students. They are training and practicing together to be a “mobile oral health team” for older adults living in a rural community 100 miles from campus. Community stakeholders are helping to organize a day-long oral health screening clinic. Looking forward, this IPE model supported by an academic-community partnership will routinely visit chronically underserved areas with a mobile oral health team. Funding for a fully-equipped mobile unit are being sought to sustain this effort.

